# Physiological-based cord clamping in very preterm infants: the Aeration, Breathing, Clamping 3 (ABC3) trial—study protocol for a multicentre randomised controlled trial

**DOI:** 10.1186/s13063-022-06789-6

**Published:** 2022-10-01

**Authors:** Ronny Knol, Emma Brouwer, Thomas van den Akker, Philip L. J. DeKoninck, Enrico Lopriore, Wes Onland, Marijn J. Vermeulen, M. Elske van den Akker–van Marle, Leti van Bodegom–Vos, Willem P. de Boode, Anton H. van Kaam, Irwin K. M. Reiss, Graeme R. Polglase, G. Jeroen Hutten, Sandra A. Prins, Estelle E. M. Mulder, Christian V. Hulzebos, Sam J. van Sambeeck, Mayke E. van der Putten, Inge A. Zonnenberg, Stuart B. Hooper, Arjan B. te Pas

**Affiliations:** 1 Division of Neonatology, Department of Paediatrics, Sophia Children’s Hospital, Erasmus MC University Medical Center, P.O. Box 2060, 3000 CB Rotterdam, The Netherlands; 2 Division of Neonatology, Department of Paediatrics, Leiden University Medical Center, Leiden, The Netherlands; 3 Department of Obstetrics, Leiden University Medical Center, Leiden, The Netherlands; 4 Athena Institute, VU University, Amsterdam, The Netherlands; 5 Department of Obstetrics and Gynaecology, Erasmus MC University Medical Center, Rotterdam, The Netherlands; 6 The Ritchie Centre, Hudson Institute of Medical Research, Monash University, Clayton, VIC Australia; 7 Department of Neonatology, Emma Children’s Hospital, Amsterdam UMC, University of Amsterdam and Vrije Universiteit Amsterdam, Amsterdam, The Netherlands; 8 Department of Biomedical Data Sciences, Leiden University Medical Center, Leiden, The Netherlands; 9 Division of Neonatology, Department of Paediatrics, Radboud University Medical Center, Radboud Institute for Health Sciences, Amalia Children’s Hospital, Nijmegen, The Netherlands; 10 Department of Neonatology, Isala Women and Children’s Hospital, Zwolle, The Netherlands; 11 Department of Paediatrics, Beatrix Children’s Hospital, University Medical Center Groningen, Groningen, The Netherlands; 12 Department of Paediatrics, Maxima Medical Center, Veldhoven, The Netherlands; 13 Department of Paediatrics, Maastricht University Medical Center, Maastricht, The Netherlands; 14 Department of Neonatology, Wilhelmina Children’s Hospital, University Medical Center Utrecht, Utrecht, The Netherlands

**Keywords:** Preterm infants, Physiological-based cord clamping, Randomised clinical trial, Study protocol, Cord clamping

## Abstract

**Background:**

International guidelines recommend delayed umbilical cord clamping (DCC) up to 1 min in preterm infants, unless the condition of the infant requires immediate resuscitation. However, clamping the cord prior to lung aeration may severely limit circulatory adaptation resulting in a reduction in cardiac output and hypoxia. Delaying cord clamping until lung aeration and ventilation have been established (physiological-based cord clamping, PBCC) allows for an adequately established pulmonary circulation and results in a more stable circulatory transition. The decline in cardiac output following time-based delayed cord clamping (TBCC) may thus be avoided. We hypothesise that PBCC, compared to TBCC, results in a more stable transition in very preterm infants, leading to improved clinical outcomes. The primary objective is to compare the effect of PBCC on intact survival with TBCC.

**Methods:**

The Aeriation, Breathing, Clamping 3 (ABC3) trial is a multicentre randomised controlled clinical trial. In the interventional PBCC group, the umbilical cord is clamped after the infant is stabilised, defined as reaching heart rate > 100 bpm and SpO_2_ > 85% while using supplemental oxygen < 40%. In the control TBCC group, cord clamping is time based at 30–60 s. The primary outcome is survival without major cerebral and/or intestinal injury. Preterm infants born before 30 weeks of gestation are included after prenatal parental informed consent. The required sample size is 660 infants.

**Discussion:**

The findings of this trial will provide evidence for future clinical guidelines on optimal cord clamping management in very preterm infants at birth.

**Trial registration:**

ClinicalTrials.gov NCT03808051. First registered on January 17, 2019.

**Supplementary Information:**

The online version contains supplementary material available at 10.1186/s13063-022-06789-6.

## Background

Infants who are born very preterm have a high mortality rate and survivors are at an increased risk of long-term neurodevelopmental sequelae and health-related problems [1, 2]. Although the care for preterm infants has improved considerably resulting in lower mortality, neonatal morbidity has not changed significantly [3]. In recent years, convincing evidence has led to novel insights regarding interventions applied for stabilisation of newborn infants during the first 10 min after birth, which may have a long-lasting impact on neonatal outcomes [4–9]. Most very preterm infants need respiratory support at birth as they fail to aerate their immature lungs independently. According to current international guidelines, the umbilical cord needs to be clamped before interventions for neonatal cardiopulmonary stabilisation can be started [10, 11]. This approach compromises cardiovascular function and placental transfusion, which paradoxically may increase the risk of mortality and morbidity [12–16]. Subsequently, more aggressive interventions may be necessary to stabilise the infant, increasing the risks of adverse outcomes.

There is strong evidence suggesting that preterm infants benefit from placental transfusion (blood transfer from the placenta to the infant) when cord clamping is delayed. Recent meta-analyses, comparing delayed cord clamping (DCC) with immediate cord clamping (ICC) in preterm infants, showed increased haematocrit, fewer blood transfusions, a decrease in mortality and a trend towards fewer intraventricular haemorrhages (IVH) [17, 18]. However, in most studies, DCC was performed using a fixed time of 30–60 s, while it can take up to 3 min before placental transfusion is complete [19]. Waiting longer than 30–60 s is not considered feasible, given that respiratory support cannot be applied during this time interval. Additionally, most trials comparing DCC to ICC did not include very preterm infants requiring immediate interventions for stabilisation or resuscitation, while these infants have the highest risk of complications and therefore could benefit most from DCC.

While the rationale of most cord clamping studies had previously been based on the effects of placental transfusion, more recent studies in preterm lambs have demonstrated that delaying cord clamping until after ventilation onset prevents a rapid decrease in cardiac output [20]. The observed large fluctuations in systemic and cerebral haemodynamics, and concomitant bradycardia and hypoxia frequently observed in preterm infants after ICC, could be avoided by delaying cord clamping until after aeration of the lung [12, 15, 21, 22]. Cardiopulmonary instability is considered an important risk factor for morbidities in preterm infants, as prolonged bradycardia and hypoxia at birth are associated with a threefold increased risk of developing IVH and death [6]. More vigorous resuscitation to correct bradycardia or hypoxia is associated with a fourfold increased risk of IVH [5]. Hypotension and assisted ventilation are associated with an increased risk of necrotising enterocolitis (NEC) and both anaemia and hypotension have been associated with an increased risk of NEC and IVH [23–25]. As a result, delaying cord clamping until the infant is stabilised may decrease the risk of cerebral injury and hypoxia-related diseases such as NEC and associated rates of mortality and morbidity [4, 6, 18].

In clinical practice, the current cord clamping approach is based on a fixed time point (time-based cord clamping (TBCC)) [10, 26]. In recent years, studies in preterm infants have been performed where respiratory support was provided prior to cord clamping. However, all these studies used a time-based approach, with cord clamping varying between 60 s and 3 min after birth [27–31]. Albeit the feasibility of the various approaches to deliver respiratory support before cord clamping was uniformly demonstrated, these trials were not adequately powered to demonstrate a difference in clinical outcomes.

Herein, we propose that optimising the perinatal stabilisation could be done by using the infant’s physiology to guide the timing of cord clamping rather than merely using a predefined time point. We have called this approach ‘physiological-based cord clamping’ (PBCC) [32]. Although no clear criteria are available to define when an infant reaches cardiorespiratory stability, the primary aim of PBCC is to ensure that the left ventricular output is maintained by achieving lung aeration and sufficient increase in pulmonary blood flow before the cord is clamped.

**Fig. 1:**
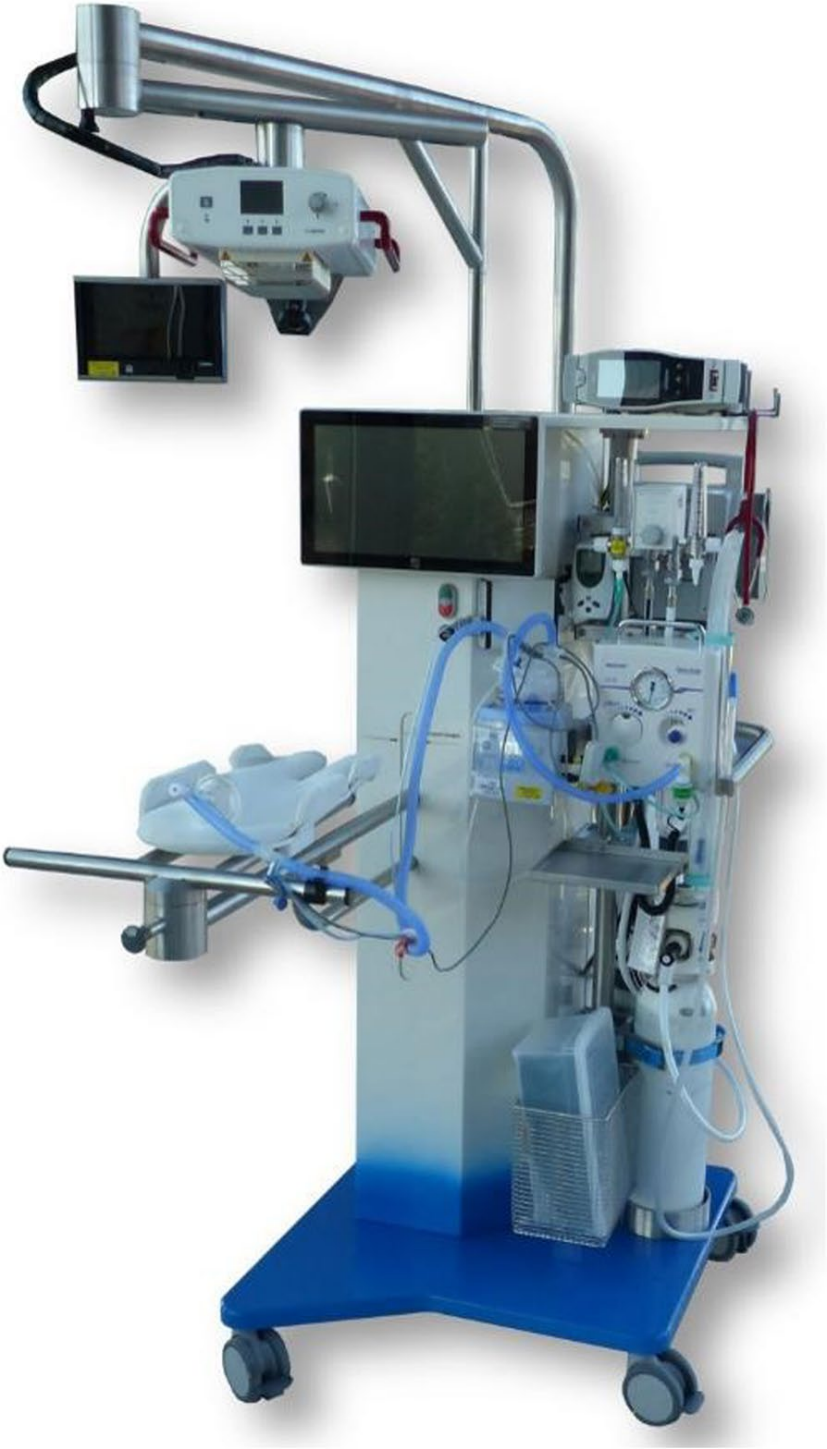
The Concord, a purpose-built resuscitation trolley developed at Leiden University Medical Centre (LUMC)

To make PBCC possible, a purpose-built resuscitation table (called the Concord) has been developed at Leiden University Medical Centre (LUMC) (Fig. 1). This mobile resuscitation trolley is designed to provide cardiorespiratory support to preterm infants at birth according to standard care while the cord remains intact. All equipment needed for stabilisation and resuscitation is incorporated into the trolley. We have described that PBCC in preterm infants using the Concord is feasible and safe [33]. Moreover, we were able to show that stabilisation of preterm infants was at least as effective as standard care [34]. In these studies, the cord was clamped if the infant had established sufficient spontaneous breathing with oxygen saturation (SpO_2_) > 90%, heart rate > 100 bpm and supplemental oxygen need < 40%. We observed less bradycardia and hypoxia at birth, confirming the more stable haemodynamic transition observed in previous preclinical studies [33]. The average time of cord clamping was more than 4 min, which may also have allowed the infants to benefit from better placental transfusion.

### Hypothesis

In this study, we will test the hypothesis that PBCC will lead to an increase in intact survival (survival without significant cerebral injury and/or NEC) when compared to TBCC.

## Methods

### Aim of the trial

The objective of the trial is to compare the effect of umbilical cord clamping after cardiopulmonary stabilisation (physiological-based cord clamping) in preterm infants on intact survival and health and non-healthcare costs to standard care (time-based cord clamping).

### Trial design

This is a multicentre randomised controlled clinical superiority trial with a parallel group design and a 1:1 allocation ratio.

### Study setting

Eligibility of patients is assessed and patients are recruited in tertiary referral centres for perinatal care, comprising an obstetric high care unit and a level 3 neonatal intensive care unit (NICU). All 9 tertiary referral centres for perinatal care in the Netherlands participate in this trial. The ABC3 study is conducted within the Neonatology Network Netherlands (N3) organisation (www.neonatology.eu).

### Study population

Eligible patients are preterm infants born at < 30 weeks of gestation in one of the participating centres after obtaining parental informed consent. Exclusion criteria are significant congenital malformations; signs of acute placental abruption; total placenta praevia, anterior placenta praevia or invasive placentation (accreta/percreta); birth by emergency caesarean section (ordered to be executed within 15 min); twin gestation with signs of twin-to-twin transfusion syndrome or twin anaemia polycythaemia syndrome not treated with fetoscopic laser treatment; multiple pregnancy > 2 (triplets or higher order); or a documented decision to give palliative neonatal care.

### Interventional treatment (PBCC)

**Fig. 2:**
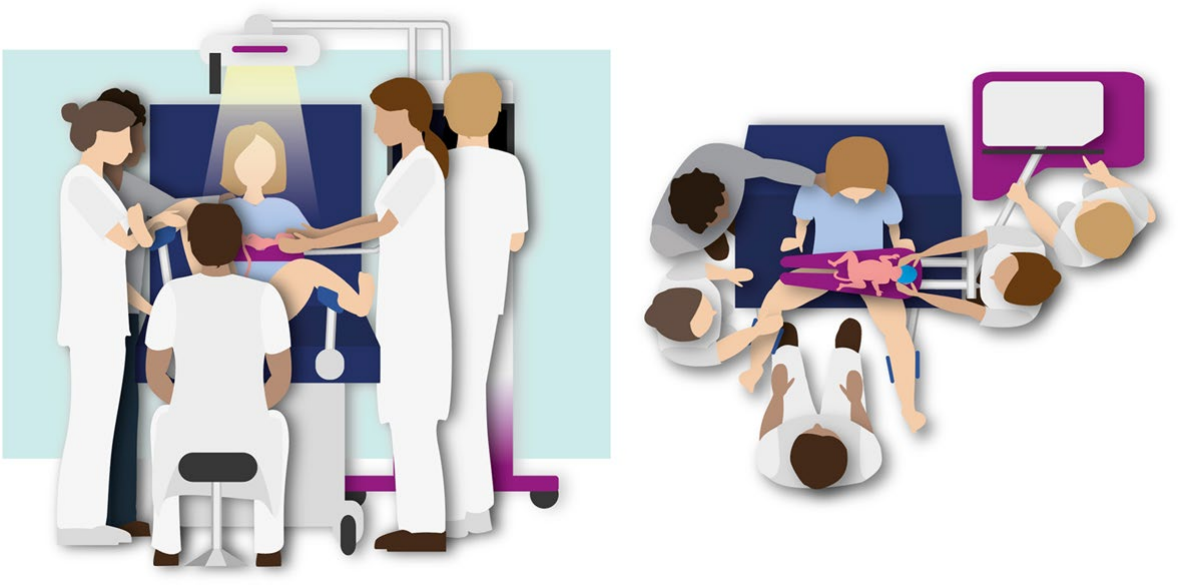
The physiological-based cord clamping procedure using the Concord, applied in the intervention group. Stabilisation of the infant is performed while the cord is intact and the cord is clamped only after the infant is stabilised

Infants randomised to the intervention group will be stabilised according to PBCC (Fig. 2). Immediately after birth, the infant will be placed on the Concord and respiratory support will be started. Temperature is managed by using a translucent wrap and radiant heater. The umbilical cord will not be clamped until the infant is stabilised. Stability is defined as reaching a heart rate > 100 bpm and SpO_2_ > 85% while using < 40% supplemental oxygen. The minimum time of cord clamping is 3 min and the maximum time is 10 min. Prior to cord clamping, a trial of weaning from positive pressure ventilation to continuous positive airway pressure is attempted. With the exception that the infant is stabilised close to the mother and the cord is clamped at a later stage, the infants will be treated according to current resuscitation guidelines. Uterotonic drugs are administered immediately after cord clamping.

### Standard treatment (TBCC)

**Fig. 3:**
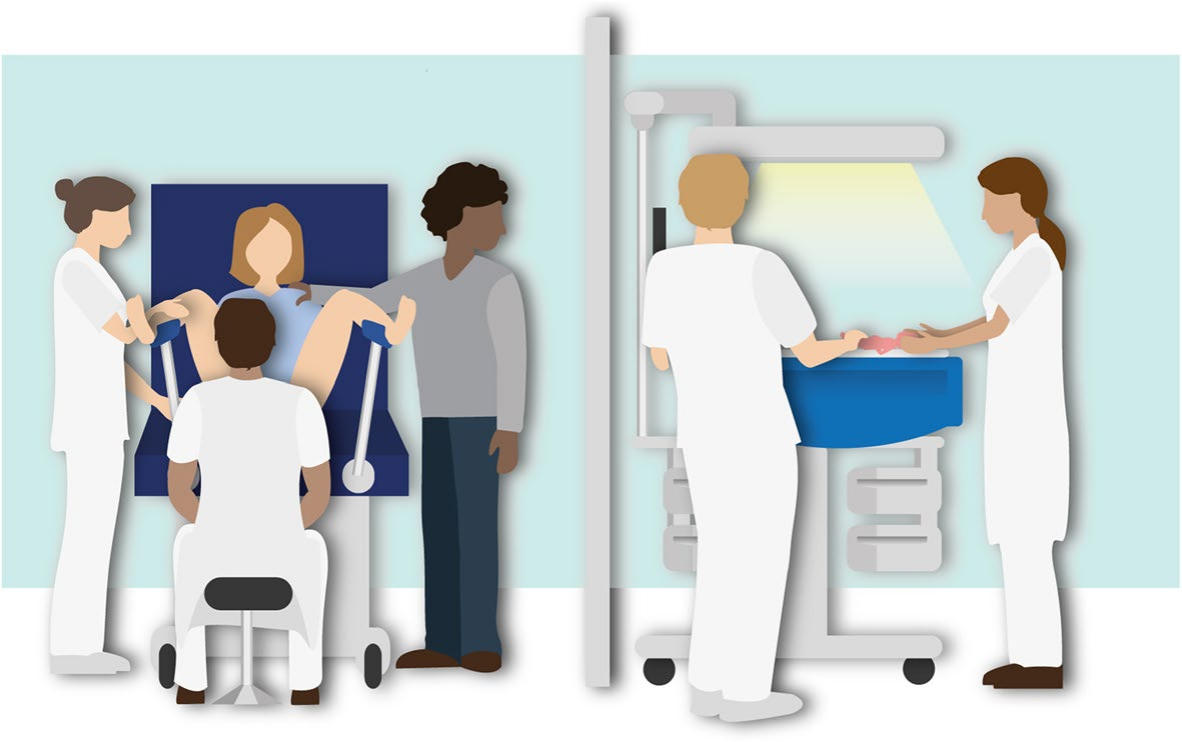
The standard time-based cord clamping procedure, applied in the control group. Cord clamping is performed immediately or delayed for 30–60 s and stabilisation of the infant is performed after the cord is clamped using a standard resuscitation table

Infants randomised to the control group will be stabilised according to the standard procedure, by clamping first and then being moved to the standard resuscitation table for further cardiopulmonary stabilisation (Fig. 3). Clamping is time based and performed immediately or delayed at 30–60 s, depending on the clinical condition of the infant. Uterotonic drugs are administered immediately after cord clamping.

### Study procedures

Prior to the start of the study, all caregivers involved in birth care will be trained in using the Concord for PBCC, for which instruction videos and workshops have been developed. A standard operating procedure has been developed for close collaboration between the obstetric and neonatal teams. All neonatal caregivers involved are trained experts and accredited for neonatal resuscitation.

All randomised patients will receive standard interventions as part of stabilisation (e.g. heat loss prevention, respiratory support). In both groups, stabilisation will be started as soon as the infant is placed on the table. The interventions are done according to international resuscitation guidelines. Small differences in standard care may exist between sites when local protocols deviate from the guidelines. All performed perinatal interventions will be recorded in the case report form (CRF).

When an infant is randomised to PBCC, the standard resuscitation table will always be prepared for use as a backup. The attending neonatologist or obstetrician can decide at any time that PBCC should not be performed or should be interrupted, after which the infant is transferred to the standard resuscitation table for (further) stabilisation.

### Investigational equipment

The Concord has been designed specifically to provide complete care to stabilise preterm infants at birth while the cord remains intact. A platform for the infant with a swivel function is placed very close to the mother. A slit in the platform protects the umbilical cord from stretching and kinking, irrespective of its length. The trolley is provided with all equipment needed for stabilisation and resuscitation. The Concords used in the first 3 participating centres are prototypes, designed and built as an investigational product by LUMC. The Concords used in all other participating centres are CE-marked birth trolleys, manufactured by the start-up company Concord Neonatal B.V. (Leiden, The Netherlands, www.concordneonatal.com).

In standard care, a standard resuscitation table is used provided with all equipment needed for stabilisation and resuscitation. This table is often situated in a separate resuscitation room. All centres are encouraged to use a respiratory function monitor, to record physiological parameters and clinical handling of the infant during stabilisation.

### Primary outcome

The primary outcome is the dichotomous outcome of intact survival at NICU discharge, defined as survival without major cerebral and/or intestinal injury (i.e. IVH ≥ grade 2 and/or PVL ≥ grade 2 and/or periventricular venous infarction; and/or NEC Bell’s stage ≥ 2).

Cerebral injury will be assessed by ultrasonography. Cerebral ultrasounds will be performed according to the national guideline at postnatal days 1, 3, 7, 14 and 28 and then every 2 weeks until NICU discharge. These scans will be performed by experienced neonatal ultrasound specialists. All cerebral ultrasound recordings will be reviewed and scored by an independent researcher blinded for the treatment allocation. For the grading of IVH and PVL, we will use the definitions of Volpe and De Vries, respectively [35, 36].

NEC will be diagnosed according to modified Bell’s staging criteria, requiring radiographical signs of pneumatosis intestinalis and/or portal venous gas to be classified as stage 2 or higher [37, 38]. The diagnosis of NEC will be ascertained by having it reviewed by an independent researcher blinded for treatment allocation. Cases of spontaneous focal intestinal perforation, defined as isolated perforation in a normal-appearing bowel without features of NEC such as pneumatosis intestinalis or necrosis, are not classified as NEC [39]. Cases without pneumatosis but with (sub)total intestinal necrosis confirmed during laparotomy or with histopathology (tissue biopsy or post-mortem) are defined as NEC stage 3 [40].

### Secondary outcomes

Demographic details and patient characteristics will be extracted from the medical files, including maternal age, parity, maternal smoking, indicators of socio-economic status, gestational age (based on known first day of last menstruation if the menstrual cycle was regular (28 days ± 5 days) or based on early foetal ultrasonography), birth weight, sex, single or twin gestations, monochorionic or dichorionic placentation, small for gestational age, mode of birth, complications of pregnancy (prelabour rupture of membranes, hypertensive disorders, chorioamnionitis, gestational diabetes), use of prenatal corticosteroids and other maternal medication. The parents will be asked to fill in a questionnaire concerning their perception and appreciation of the approach during birth and the perinatal stabilisation.

Various secondary outcomes are collected during the NICU stay and after discharge until the corrected age of 2 years. All clinical secondary endpoints are measured as standard care and will be extracted from the medical charts of the patients by the investigators.

#### Procedure related

Details of the stabilisation at birth (cord clamping time) and interventions (respiratory support, maximum supplemented oxygen); treatment failure defined as abortion of prescribed procedure (intervention or control) and reasons for abortion; infant temperature at NICU admission; highest infant haemoglobin level within 24 h of age; polycythaemia (venous haematocrit > 0.65).

#### Neonatal outcomes, short-term

Apgar scores; intubation in the first 72 h; respiratory distress syndrome; use of surfactant; intravascular volume expansion in the first 72 h; cardiovascular medication use in the first 72 h; pneumothorax; persistent ductus arteriosus for which medical intervention or surgical ligation is indicated; highest bilirubin level; phototherapy and/or exchange transfusion for hyperbilirubinemia; proven early- and late-onset sepsis; NEC; spontaneous focal intestinal perforation; number of red blood cell transfusions; IVH; periventricular leukomalacia; periventricular venous infarction; bronchopulmonary dysplasia at 36 weeks postmenstrual age (PMA), graded according to NICHD criteria [41, 42]; retinopathy of prematurity; mortality at 28 days postnatal age, 36 weeks PMA and at hospital discharge; length of NICU stay; and length of hospital stay.

#### Maternal outcomes

Estimated total blood loss; postpartum haemorrhage > 1000 mL; placental weight; surgical site infection after caesarean section.

#### Neonatal outcomes, long-term

Long-term neurodevelopmental outcomes assessed at 2 years corrected age with Bayley Scales of Infant Development III (BSID-III-NL); Mental Developmental Index (MDI); Psychomotor Developmental Index (PDI); cerebral palsy and severity; hearing loss requiring hearing aids; blindness; and behavioural problems.

#### Quality of life of children and parents

Quality of life of children will be assessed using the TNO-AZL Preschool Children’s Quality of Life (TAPQOL) questionnaire and the Pediatric Quality of Life Inventory (PedsQL; generic score, adapted for young children) questionnaire. Quality of life of parents is measured using the EQ-5D-5L questionnaire. The TAPQOL and EQ-5D-5L will be completed at 6 months corrected age, and subsequently every 6 months during follow-up until 2 years corrected age. The PedsQL (generic score) questionnaire will be completed at 18 months and 2 years corrected age.

#### Healthcare and non-healthcare costs

Healthcare costs include all healthcare use during follow-up, e.g. NICU days, readmissions, treatments, outpatient visits and general practitioner visits. Non-healthcare costs consist of lost productivity costs of parents from paid and unpaid work and costs of (specialised) daycare for children. Healthcare use and absence from work will be assessed by parents every 6 months.

**Fig. 4:**
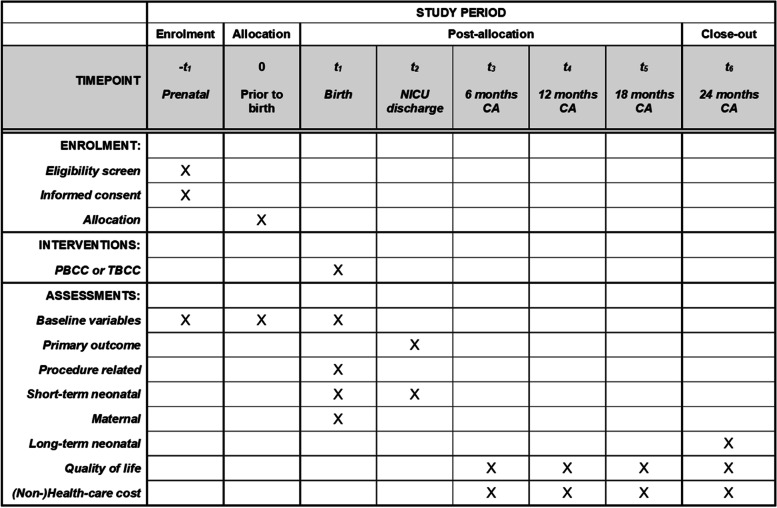
Timeline schedule of eligibility screening, consent, enrolment, allocation, intervention and assessments at all time points. *CA* corrected age

The SPIRIT 2013 statement, 33-item checklist (additional file supplemented) and figure (Fig. 4), is being used to schematically represent the study participants’ timeline of eligibility screening, enrolment, allocation, intervention and assessments at all time points and to guide the overall standards of the study [43].

### Sample size calculation

Estimation of the background incidence of the primary outcome in preterm infants below 30 weeks of gestation is estimated at 72%, based on historical databases of LUMC and Erasmus MC pertaining to recent years. Estimation of the effect size of the intervention cannot be based on earlier trials, as this will be the first human clinical trial on efficacy.

We estimated the possible effect of the intervention based on the following arguments:

- In the preclinical and the feasibility studies, PBCC has led to less bradycardia, less cerebral hypoperfusion, fewer fluctuations in cardiac output and less hypoxia at birth, which may all be related to the primary outcome [12, 15, 20, 33].
- A high-risk population of preterm infants is studied, who usually need stabilisation at birth. These infants may benefit most from PBCC and placental transfusion.
- Using the PBCC approach allows for more complete placental transfusion, which may enhance the beneficial effects as seen earlier in the DCC versus ICC trials. It was shown in a recent meta-analysis that mortality was significantly reduced (relative risk (RR) 0.68, [95% confidence interval (CI) 0.52, 0.90]) and there was a trend towards a lower incidence of IVH (RR 0.87 [95% CI 0.75, 1.00]) [17]. We expect a larger effect of PBCC as compared to DCC because in most DCC studies cord clamping was performed at 30 to 60 s and infants requiring resuscitation or stabilisation were excluded.

**Fig. 5:**
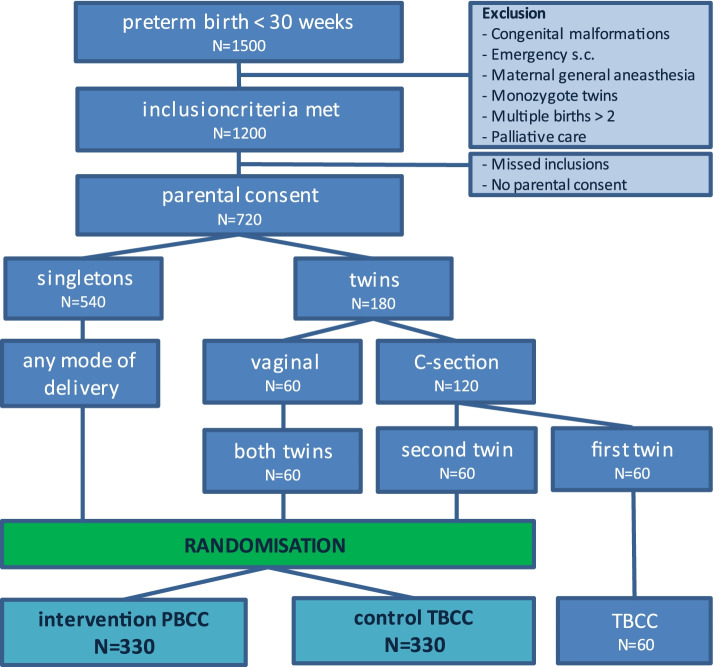
Flow chart illustrating the randomisation plan showing expected numbers needed to include 330 participants in each arm

Taking these numbers into account, we consider an absolute increase of 10% of the intact survival (from 72 to 82%) to be a realistic and clinically relevant effect. We calculated that at least 550 (275 in each arm) infants are needed to detect an absolute difference of 10% (0.72 to 0.82 intact survival), with 80% power, at a significance level of 0.05. To correct for twins (one twin pair assumed equally informative as one singleton participant; in case of caesarean section, first twins are not randomised) and an anticipated 10% crossover from the interventional group to the standard group, the sample size was increased to 330 participants in each arm. In Fig. 5, the expected numbers needed to achieve this goal are illustrated.

### Recruitment and consent

Eligible patients are recruited at the obstetrics ward. All women at risk of preterm birth prior to 30 weeks of gestation, either spontaneous or iatrogenic, are screened. Prenatal written informed consent will be obtained if the woman is not in established labour and if time permits. In this situation, parents of an eligible infant will be informed by the local investigator, the attending obstetrician or the attending neonatologist and asked for their consent after they have read the information letter. To limit selection bias and increase generalisability, we will strive to also include the most unexpected born preterm infants in the trial. For this reason, we will also approach parents in case the woman arrives in the hospital in full labour. Parents will be informed on study goals and procedures and asked for oral consent. Written informed consent will be obtained as soon as possible afterwards.

We will not approach parents for consent in case of an emergency situation and immediate birth (< 15 min) is necessary or when approaching parents for consent is considered inappropriate. These infants will not be included in the study and deferred consent will not be used.

### Randomisation, blinding and treatment allocation

Infants will be 1:1 randomised to either PBCC or standard treatment. Allocation will be stratified by gestational age (24–26 + 6 and 27 –29 + 6 weeks) and by treatment centre using random permutated block (4–8) sizes. Concealment of allocation will be ensured by using the randomisation process of Castor Electronic Data Capture (Amsterdam, The Netherlands, www.castoredc.com), an electronic data capture system. Blinding of the allocation arm during the intervention is not possible in this trial. Independent assessors who verify the primary outcome are blinded for treatment allocation.

In case of twin vaginal birth, both infants will be randomised to the same group. In case of twin caesarean section, it is technically not possible at this moment to perform PBCC in both infants. After consent, both infants will be included; the first infant will always receive standard treatment and the second infant will be randomised to either PBCC or standard treatment.

### Withdrawal of subjects

Parents and caregivers can leave the study at any time for any reason if they wish to do so without any consequences. If consent is withdrawn before NICU discharge, replacement will take place by inclusion and randomisation of another infant.

The clinician can decide to stop the PBCC approach for urgent medical reasons and switch to standard care. As early abortion of PBCC is a secondary outcome parameter, these infants are not withdrawn from the study but remain in follow-up. PBCC can be aborted immediately, when:

- An emergency occurs with the mother or the second twin and more working space is needed for the obstetric team.
- Full cardiac resuscitation for the infant is needed.
- Maternal blood loss is excessive according to the obstetric team and immediate administration of uterotonic drugs is necessary.

### (Serious) adverse event reporting (SAE)

This study population has a high risk of serious complications (so-called context-specific SAE’s), which are inherent to their vulnerable condition and unrelated to the intervention which is under evaluation in this trial. Immediate and individual reporting of all these condition-related complications will not enhance the safety of the study. These complications are included in the primary and secondary outcomes of this study and are recorded during NICU admission in the CRF. This documentation will include the date of diagnosis, classification/gradation of the complication and type of action taken if appropriate. Yearly, an overview of the context-specific SAEs for each treatment arm will be presented to the Data safety Monitoring Committee (DMC) and Medical Research Ethics Committee (MREC).

Stabilisation of preterm infants with the resuscitation table as close as possible to the mother has been performed before and is considered safe. We do not expect PBCC to pose additional risks for the infant compared to the risks related to stabilisation of a preterm infant. In addition to infant mortality during NICU admission, we will include three ‘safety parameters’ as SAE in this study that will be reported to the MREC after obtaining knowledge of the event:

- Severe hypothermia at NICU admission (defined by WHO as temperature < 32° C)
- Severe maternal postpartum haemorrhage (defined as blood loss > 1000 mL)
- Rupture of the umbilical cord

All SAEs will be reported by the principal investigator to the accredited MREC that approved the protocol. Any unforeseen SAE that was life threatening or resulted in death and was directly related to the PBCC approach will be reported to the MREC without undue delay after obtaining knowledge of the event.

Any unforeseen SAE directly related to the PBCC approach and not considered life threatening or resulting in death are recorded in the CRF and included in the yearly overview of the context-specific SAEs that will be presented to the DMC and MREC.

All SAEs that are derived from the medical charts of the patients, which do not meet the previously outlined criteria of a SAE related to the PBCC approach and the context-specific SAEs, are recorded in the CRF and included in the yearly overview of the context-specific SAEs that will be presented to the DMC and MREC.

### Statistical analysis

A detailed Statistical Analysis Plan will be published separately towards the end of the inclusion period. Intention-to-treat analysis will be employed as the primary analysis and as-treated analyses as secondary. The effect of PBCC on the primary and secondary outcomes will be assessed by multi-variable logistic regression analysis, taking the stratifying factors and potential correlation between siblings into account. The interim analyses will include the assessment of the effect of PBCC on the combined primary outcome and its components, as well as the effect on the predefined safety outcomes maternal blood loss, infant hypothermia and umbilical cord rupture. Statistical significance is set at *p* < 0.05.

### Cost-effectiveness analysis

The economic evaluation from a societal perspective will consist of a trial-based cost-effectiveness analysis (costs per additional infant with intact survival) and a model-based cost-utility analysis (lifelong costs per QALY).

In the trial-based economic evaluation, the effects (in terms of quality-adjusted life years (QALYs)) of PBCC will be compared to TBCC and related to the difference in costs during the follow-up period of 2 years. Costs consist of healthcare costs and non-healthcare costs. Healthcare use will be multiplied by Dutch reference prices to obtain healthcare costs [44]. Non-healthcare costs will be obtained by using the friction cost method for absenteeism of paid work by parents, valuing the lost hours of unpaid work by their opportunity costs and (specialised) daycare by its market price.

QALYs will be assessed using the quality of life questionnaires (EQ-5D-5L, PedsQL and TAPQOL). From these questionnaires, utilities will be calculated using so-called tariffs (EQ-5D-5L for parents) and the indirect mapping approach for the PedsQL and TAPQOL (for infants). By applying the area-under-the-curve method for the utility scores obtained for infants and parents, QALYs for infants and parents will be obtained.

Differences in mean costs and effects between strategies will be compared with two-sided bootstrapping. In a net-benefit analysis, costs will be related to the outcomes and presented in a cost-effectiveness acceptability curve. No discounting will be applied due to the short time horizon of the trial-based economic evaluation. For the cost-effectiveness analysis, multiple imputation will be used for handling missing data.

### Data handling and study monitoring

Data management will be implemented according to Good Clinical Practice (GCP) guidelines. Patient data will be entered by way of an electronic CRF in a central GCP proof Internet-based database to facilitate on-site data entry (Castor Electronic Data Capture, www.castoredc.com). Security is guaranteed with login names, login codes and encrypted data transfer. An experienced data manager will maintain the database and check the information in the database for completeness, consistency and plausibility.

The data of all subjects will be coded and this coding will not be retraceable to the individual patient. The key to this coding is safeguarded by the investigator. A limited number of people have access to the source data. These are the principal investigators and investigating personnel. Personal data are only processed by the researchers or by those who fall directly under their authority. In addition, the study monitor, quality assurance auditor, employees from the MREC and the Health Care Inspectorate of the Ministry of Health have access to the source data. All are subject to the pledge of confidentiality. Data will be stored for 15 years strictly confidential.

The study will be monitored by a certified monitor throughout its duration by means of personal visits to the investigator’s facilities and through other communications (e.g. telephone calls, written correspondence). These visits will be conducted to evaluate the progress of the study, to ensure the rights and wellbeing of the subjects are protected and to check that the reported clinical study data are accurate, complete and verifiable from source documents and that the conduct of the study complies with the approved protocol and amendments, GCP and applicable national regulatory requirements. A monitoring visit will include a review of the essential clinical study documents (regulatory documents, CRFs, source documents, subject informed consent forms, etc.) as well as discussion on the conduct of the study with the investigator and staff.

### Ethical considerations

The study will be conducted according to the principles of the Declaration of Helsinki and in accordance with the Dutch law (Medical Research Involving Human Subjects Act). Delayed cord clamping has been incorporated in international guidelines, mostly using a fixed time and delaying stabilisation until the cord has been clamped. So far, stabilisation with the cord intact has been considered a safe approach, following vaginal birth as well as caesarean section. The infant potentially benefits more from delaying cord clamping when PBCC is used. We do not expect an additional risk of the PBCC approach as the Concord is fully equipped for stabilisation and resuscitation. While the parents may benefit from having their baby close and being able to touch the infant, there is a risk that it will cause anxiety as interventions take place close to them. We will minimise this risk by prenatally communicating to the parents what to expect. Parental appreciation of the approach is included as a secondary outcome.

### Trial Steering Committee

The Trial Steering Committee (TSC) is the main policy and decision-making committee of the study and has final responsibility for the scientific conduct of the study. The TSC will provide overall supervision of the trial and will ensure that the trial is being conducted in accordance with the principles of GCP and the relevant regulations. The TSC is composed of representatives of the initiating centre and of investigators of the participating centres.

### Data safety Monitoring Committee and interim analyses

An external DMC will monitor safety outcomes and will provide the TSC with recommendations regarding the continuation or premature termination of the trial (for all patients or subgroups of patients). The safety data will include, but will not be restricted to, serious adverse events and the safety outcomes listed as secondary outcomes. The DMC will not be blinded to the treatment allocation. Two interim statistical analyses will be conducted on safety during the course of this study, after approximately 25% and 50% of the total required patients have completed their primary outcome. The results of the interim analyses will be assessed by the DMC, which will act completely independently of the clinical investigators, including the principal investigators. If the DMC recommends modification of the protocol or cessation of the study, this will be discussed with the TSC, who will be responsible for the final decision.

The advice(s) of the DMC will only be sent to the initiating centre of the study. Should the initiating centre and TSC decide not to fully implement the advice of the DMC, the initiating centre will send the advice to the reviewing MREC, including a note to substantiate why (part of) the advice of the DMC will not be adhered to.

### Communication of important protocol amendments

All substantial amendments will be notified to the MREC and to the competent authority. Non-substantial amendments will not be notified to the accredited MREC and the competent authority, but will be recorded and filed by the initiating centre.

### Dissemination of results

Results of the study will be presented in multiple manuscripts, which will be submitted for publication in peer-reviewed international medical journals. The results will also be presented at international conferences. Additionally, study results will be used to inform local, national and international resuscitation guidelines. News letters will be used for feedback on trial results to participating parents.

## Discussion

The aim of this trial is to assess the effect of PBCC on intact survival in very preterm infants. Performing PBCC in preterm infants will establish lung aeration and trigger the rapid increase in pulmonary blood flow and pulmonary gas exchange prior to umbilical cord clamping. Experimental studies in animals have already shown increased haemodynamic stability when using the PBCC approach [12, 15]. In our clinical approach, the cord is clamped when the infant is considered respiratory stable, but clear criteria for respiratory stability are lacking. In this study, we defined stability as reaching a heart rate > 100 bpm and SpO_2_ > 85% while using supplemental oxygen < 40%. The oxygen saturation target is set 5% lower compared to our previous clinical studies, as current international guidelines aim for an SpO_2_ of 85% at 5 min [10]. Others have used different parameters to define respiratory stability in PBCC, such as exhaled carbon dioxide as a marker for pulmonary gas exchange [31]. Since our definition performed well in our previous clinical studies and oxygen saturation and heart rate are uniformly monitored for every very preterm infant, we are confident that the criteria used in this study reflect lung aeration, pulmonary blood flow and gas exchange.

In general, obstetricians might be hesitant to delay cord clamping, mainly as this potentially could result in increased maternal blood loss. Restricting maternal blood loss is an important goal of obstetric care [45]. However, in previous studies where DCC was performed no increase in maternal blood loss was observed [18, 46]. Although cord clamping is performed later in our PBCC group than in previously reported DCC studies in very preterm infants, maternal blood loss was not observed to have increased in this group in our previous studies [33, 34]. Maternal blood loss and the incidence of postpartum haemorrhage will be important outcomes in the ABC3 trial and will be recorded as SAE.

The Concord was specifically developed and designed to perform PBCC. All necessary interventions to stabilise the infant according to international resuscitation guidelines can be provided, heart rate and SpO_2_ can be monitored, stabilisation is possible when the cord is very short (without stretching or kinking the cord), and there is little interference in terms of space between the neonatal and the obstetric healthcare providers. Training of involved caregivers using the equipment is essential, as well as briefings and debriefings before and after the procedures. Obviously, blinding of parents and caregivers to the intervention is not possible in this trial. Deferred consent was not considered appropriate in this trial, but the possibility of prenatal oral consent aims to also include women giving birth soon after admittance in hospital, thereby increasing the generalisability of trial results while respecting the needs of the parents.

The main hypothesis of the ABC3 trial is that PBCC results in optimal placental transfusion and cardiopulmonary stability during the transition of intra- to extrauterine life, leading to improved clinical outcomes. The logical challenge of stabilising preterm infants with an intact umbilical cord is that it needs to be performed very close to the mother. Preventing the infant from being separated from the mother directly after birth may be an additional advantage of the PBCC approach [47, 48]. Though this is not the primary focus of the approach, data on how parents experienced the process of birth and stabilisation of their infant will be collected by standardised questionnaires and explored. The results of this trial will be used to inform local, national and international resuscitation guidelines.

### Trial status

The first patient in the ABC3 trial was included on January 25, 2019, at the Leiden University Medical Centre. Three extra sites started recruiting during 2019, three more sites started in 2020 and the last two sites in 2021. Full recruitment will be expected in late 2022.

## Supplementary Information

**Additional file 1.** SPIRIT checklist for *Trials*.

## Data Availability

Study data from the trial described in this manuscript will be retained and archived for a minimum of 15 years after study completion as per national regulations. There are no plans for publicly sharing the trial data. All data generated and/or analysed during the trial are available from the corresponding author on reasonable request.

## Abbreviations

ABC: Aeration, Breathing, Clamping
DCC: Delayed cord clamping
ICC: Immediate cord clamping
IVH: Intraventricular haemorrhage
NEC: Necrotising enterocolitis
PVL: Periventricular leukomalacia
TBCC: Time-based cord clamping
PBCC: Physiological-based cord clamping
LUMC: Leiden University Medical Centre
NICU: Neonatal intensive care unit
CRF: Case report form
QALY: Quality-adjusted life year
(S)AE: (Serious) adverse event
DMC: Data safety Monitoring Committee
MREC: Medical Research Ethics Committee
WHO: World Health Organization
GCP: Good Clinical Practice
TSC: Trial Steering Committee
